# Unusual Spin Exchanges Mediated by the Molecular Anion P_2_S_6_^4−^: Theoretical Analyses of the Magnetic Ground States, Magnetic Anisotropy and Spin Exchanges of MPS_3_ (M = Mn, Fe, Co, Ni)

**DOI:** 10.3390/molecules26051410

**Published:** 2021-03-05

**Authors:** Hyun-Joo Koo, Reinhard Kremer, Myung-Hwan Whangbo

**Affiliations:** 1 Department of Chemistry and Research Institute for Basic Sciences, Kyung Hee University, Seoul 02447, Korea; 2 Max Planck Institute for Solid State Research, Heisenbergstrasse 1, D-70569 Stuttgart, Germany; rekre@mpg.fkf.de; 3 Department of Chemistry, North Carolina State University, Raleigh, NC 27695-8204, USA

**Keywords:** magnetic ground state, spin exchange, magnetic anisotropy, molecular anion, MPS_3_, magnetic orbitals, qualitative rules

## Abstract

We examined the magnetic ground states, the preferred spin orientations and the spin exchanges of four layered phases MPS_3_ (M = Mn, Fe, Co, Ni) by first principles density functional theory plus onsite repulsion (DFT + U) calculations. The magnetic ground states predicted for MPS_3_ by DFT + U calculations using their optimized crystal structures are in agreement with experiment for M = Mn, Co and Ni, but not for FePS_3_. DFT + U calculations including spin-orbit coupling correctly predict the observed spin orientations for FePS_3_, CoPS_3_ and NiPS_3_, but not for MnPS_3_. Further analyses suggest that the ||z spin direction observed for the Mn^2+^ ions of MnPS_3_ is caused by the magnetic dipole–dipole interaction in its magnetic ground state. Noting that the spin exchanges are determined by the ligand p-orbital tails of magnetic orbitals, we formulated qualitative rules governing spin exchanges as the guidelines for discussing and estimating the spin exchanges of magnetic solids. Use of these rules allowed us to recognize several unusual exchanges of MPS_3_, which are mediated by the symmetry-adapted group orbitals of P_2_S_6_^4−^ and exhibit unusual features unknown from other types of spin exchanges.

## 1. Introduction

In an extended solid, transition-metal magnetic cations M are surrounded by main-group ligands L to form ML_n_ (typically, *n* = 3–6) polyhedra, and the unpaired spins of M are accommodated in the singly occupied d-states (i.e., the magnetic orbitals) of ML_n_. Each d-state has the metal d-orbital combined out-of-phase with the p-orbitals of the surrounding ligands L. The tendency for two adjacent magnetic ions to have a ferromagnetic (FM) or an antiferromagnetic (AFM) spin alignment is determined by the spin exchange between them, which takes place through the M-L-M or M-L…L-M exchange path [1,2,3,4]. Whereas the characteristics (e.g., the angular and distance dependence) of the M-L-M exchanges is conceptually well understood [5,6,7,8], the properties of the M-L…L-M exchanges involving several main-group ligands have only come into focus in the last two decades [1,2,3,4]. Furthermore, the character of a M-L…L-M exchange can be modified if the L…L contact is bridged by a d^0^ metal cation A to form a L…A…L bridge [1,2,3,4]. What has not been well understood so far is the M-L…L-M exchange in which the L…L contact is an integral part of the covalent framework of a molecular anion made up of main group elements (e.g., the P_2_S_6_^4−^ anion in MPS_3_, where M = Mn, Fe, Co, Ni), which might be termed the M-(L-L)-M exchange to emphasize its difference from the M-L-M, M-L…L-M and M-L…A…L-M exchanges.

**Figure 1:**
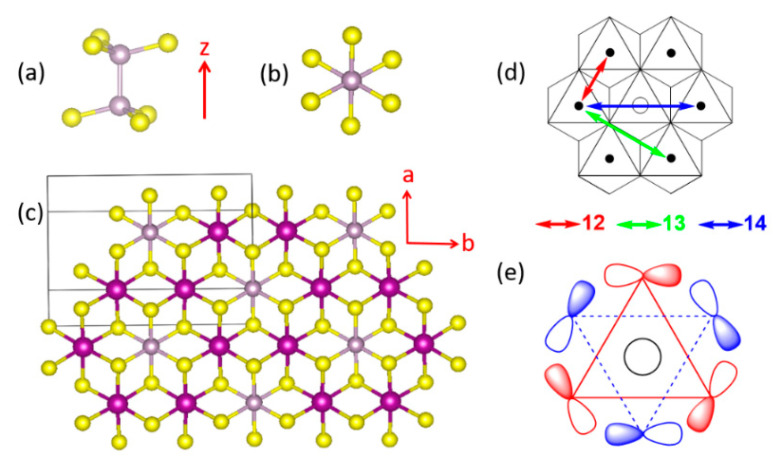
(**a**) Perspective and (**b**) projection views of a P_2_S_6_^4−^ anion. (**c**) A projection view of a single MPS_3_ layer along the c*-direction (i.e., the z-direction), which is perpendicular to the MPS_3_ layer. (**d**) Three kinds of the spin exchange paths in the MPS_3_ honeycomb layers of MPS_3_, where the labels 12, 13 and 14 refer to J_12_, J_13_ and J_14_, respectively. (**e**) A group orbital of P_2_S_6_^4−^ viewed along the P-P axis. The red triangle represents the three S atoms of the upper PS_3_ pyramid, and the blue triangles those of the lower PS_3_ pyramid.

In the present work we examine the M-(L-L)-M spin exchanges in the layered phases MPS_3_ (M = Mn [9,10,11], Fe [9,10,11], Co [10,11], Ni [10,11]), which crystallize with a monoclinic structure (space group *C*2/*m*, no. 12). Each layer of MPS_3_ is made up of the molecular anions P_2_S_6_^4−^ possessing the structure of staggered ethane (i.e., a trigonal antiprism structure) (Figure 1a,b). The molecular anions P_2_S_6_^4−^ form a trigonal layer (Figure 1c) with the P-P bonds perpendicular to the layer, and a high-spin M^2+^ cation occupies every S_6_ octahedral site (deviations from a trigonal symmetry caused by the monoclinic distortions are less than 1°). Thus, each MPS_3_ layer consists of a honeycomb arrangement of M^2+^ cations. With the c*-direction of the MPS_3_ taken as the z-direction, the P-P bond of each P_2_S_6_^4−^ is parallel to the z-direction (||z), and each MS_6_ octahedron is arranged with one of its three-fold rotational axes along the ||z-direction.

To a first approximation, it may be assumed that each MPS_3_ layer has a trigonal symmetry (see below for further discussion), so there are three types of spin exchanges to consider, i.e., the first nearest-neighbor (NN) spin exchange J_12_, the second NN spin exchange J_13_, and the third NN exchange J_14_ (Figure 1d). J_12_ is a spin exchange of the M-L-M type, in which the two metal ions share a common ligand, while J_13_ and J_14_ are nominally spin exchanges of the M-L…L-M type, in which the two metal ions do not share a common ligand. In describing the magnetic properties of MPS_3_ in terms of the spin exchanges J_12_, J_13_ and J_14_, an interesting conceptual problem arises. Each P_2_S_6_^4−^ anion is coordinated to the six surrounding M^2+^ cations simultaneously (Figure 1c,d), so one P_2_S_6_^4−^ anion participates in all three different types of spin exchanges simultaneously with the surrounding six M^2+^ ions. Furthermore, the lone-pair orbitals of the S atoms of P_2_S_6_^4−^, responsible for the coordination with M^2+^ ions, form symmetry-adapted group orbitals, in which all six S atoms participate (for example, see Figure 1e). Consequently, there is no qualitative argument with which to even guess the possible differences in J_12_, J_13_, and J_14_. Over the past two decades, it became almost routine to quantitatively determine any spin exchanges of a magnetic solid by performing an energy-mapping analysis based on first principles DFT calculations. From a conceptual point of view, it would be very useful to have qualitative rules with which to judge whether the spin exchange paths involving complex intermediates are usual or unusual.

**Figure 2:**
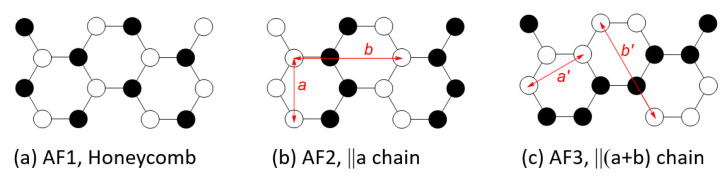
(**a**) The honeycomb AFM state, AF1. (**b**) The ||a-chain AFM state, AF2. (**c**) The ||(*a* + *b*)-chain AFM state, AF3.

A number of experimental studies examined the magnetic properties of MPS_3_ (M = Mn [9,11,12,13,14], Fe [9,11,15,16,17,18], Co [11,19], Ni [11,20]). The magnetic properties of MPS_3_ (M = Mn, Fe, Co, Ni) monolayers were examined by DFT calculations to find their potential use as single-layer materials possessing magnetic order [21]. The present work is focused on the magnetic properties of bulk MPS_3_. For the ordered AFM states of MPS_3_, the neutron diffraction studies reported that the layers of MnPS_3_ exhibits a honeycomb-type AFM spin arrangement, AF1 (Figure 2a), but those of FePS_3_, CoPS_3_ and NiPS_3_ a zigzag-chain spin array, AF2 (Figure 2b), in which the FM chains running along the a-direction are antiferromagnetically coupled (hereafter, the ||*a*-chain arrangement). An alternative AFM arrangement, AF3 (Figure 2c), in which the FM zigzag chains running along the (*a* + *b*)-direction are antiferromagnetically coupled (hereafter, the ||(*a* + *b*)-chain arrangement), is quite similar in nature to the ||a-chain arrangement.

At present, it is unclear why the spin arrangement of MnPS_3_ differs from those of FePS_3_, CoPS_3_ and NiPS_3_ and why FePS_3_, CoPS_3_ and NiPS_3_ all adopt the ||a-chain arrangement rather than the ||(*a* + *b*)-chain arrangement. To explore these questions, it is necessary to examine the relative stabilities of a number of possible ordered spin arrangements of MPS_3_ (M = Mn, Fe, Co, Ni) by electronic structure calculations and analyze the spin exchanges of their spin lattices.

Other quantities of importance for the magnetic ions M of an extended solid are the preferred orientations of their magnetic moments with respect to the local coordinates of the ML_n_ polyhedra. These quantities, i.e., the magnetic anisotropy energies, are also readily determined by DFT calculations including spin orbit coupling (SOC). For the purpose of interpreting the results of these calculations, the selection rules for the preferred spin orientation of ML_n_ were formulated [2,3,22,23,24] based on the SOC-induced interactions between the highest-occupied molecular orbital (HOMO) and lowest-unoccupied molecular orbital (LUMO) of ML_n_. With the local z-axis of ML_n_ taken along its *n*-fold rotational axis (*n* = 3, 4), the quantity needed for the selection rules is the minimum difference, |Δ*L*_z_|, in the magnetic quantum numbers *L*_z_ of the d-states describing the angular behaviors of the HOMO and LUMO. It is of interest to analyze the preferred spin orientations of the M^2+^ ions in MPS_3_ (M = Mn, Fe, Co, Ni) from the viewpoint of the selection rules.

Our work is organized as follows: Section 2 describes simple qualitative rules governing spin exchanges. The details of our DFT calculations are presented in Section 3.1. The magnetic ground states of MPS_3_ (M = Mn, Fe, Co, Ni) are discussed in Section 3.2, the preferred spin orientations of M^2+^ ions of MPS_3_ in Section 3.3, and the quantitative values of the spin exchanges determined for MPS_3_ in Section 3.4. We analyze the unusual features of the calculated spin exchanges via the P_2_S_6_^4−^ anion in Section 3.5, and investigate in Section 3.6 the consequences of the simplifying assumption that the honeycomb spin lattice has a trigonal symmetry rather than a slight monoclinic distortion found experimentally. Our concluding remarks are summarized in Section 4.

## 2. Qualitative Rules Governing Spin Exchanges

### 2.1. Spin Exchange between Magnetic Orbitals

For clarity, we use the notation $\left(\varphi_{i},\varphi_{j}\right)$ to represent the spin exchange arising from the magnetic orbitals $\varphi_{i}$ and $\varphi_{j}$ at the magnetic ion sites A and B, respectively. It is well known that $\left(\varphi_{i},\varphi_{j}\right)$ consists of two competing terms [1,2,3,4,25]

(1) $$(\varphi_{i},\varphi_{j})=J_{F}+J_{AF}$$

The FM component $J_{F}$ (>0) is proportional to the exchange repulsion,

(2) $$J_{F}\propto K_{ij}$$

which increases with increasing the overlap electron density $\rho_{ij}=\varphi_{i}\varphi_{j}$. In case when the magnetic orbitals $\varphi_{i}$ and $\varphi_{j}$ are degenerate (e.g., between the t_2g_ states or between e_g_ states of the magnetic ions at octahedral sites), the AFM component J_AF_ (<0) is proportional to the square of the energy split $\Delta e_{ij}$ between $\varphi_{i}$ and $\varphi_{j}$ induced by the interaction between them,

(3) $$J_{AF}\propto -{\left(\Delta e_{ij}\right)}^{2}\propto -{\left(S_{ij}\right)}^{2}$$

The energy split $\Delta e_{ij}$ is proportional to the overlap integral $S_{ij}$ = 〈$\varphi_{i}$|$\varphi_{j}$〉, so that the magnitude of the AFM component $J_{AF}$ increases with increasing that of ${\left(S_{ij}\right)}^{2}$. If $\varphi_{i}$ and $\varphi_{j}$ are not degenerate (e.g., between the t_2g_ and e_g_ states of the magnetic ions), the magnitude of $J_{AF}$ is approximately proportional to $-{\left(S_{ij}\right)}^{2}$.

### 2.2. p-Orbital Tails of Magnetic Orbitals

**Figure 3:**
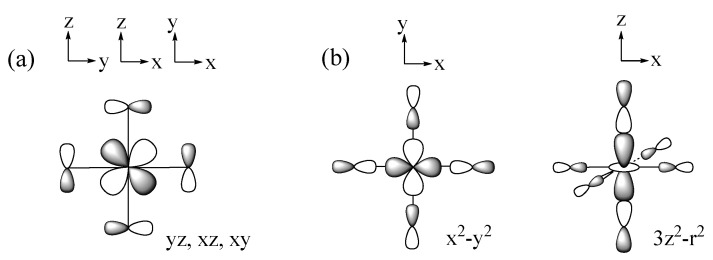
(**a**) The t_2g_ states and (**b**) the e_g_ states of a ML_6_ octahedron.

**Figure 4:**
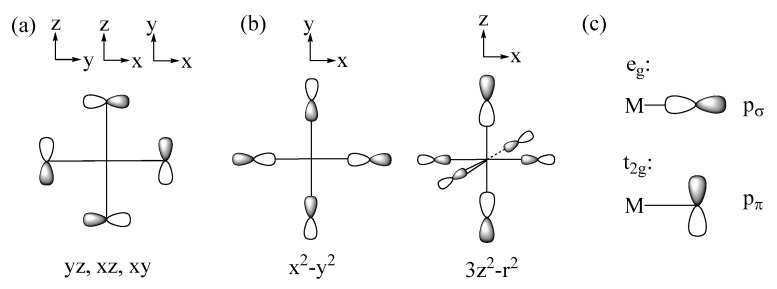
The p-orbital tails of (**a**) the t_2g_ and (**b**) the e_g_ states of a ML_6_ octahedron. (**c**) The p_σ_ and p_π_ orbitals of the ligand p-orbital tails.

The spin exchanges between adjacent transition-metal cations M are determined by the interactions between their magnetic orbitals, which in turn are governed largely by the overlap and the overlap electron density that are generated by the p-orbitals of the ligands present in the magnetic orbitals (the p-orbital tails, for short) [1,2,3,4]. Suppose that the metal ions M are surrounded by main-group ligands L to form ML_6_ octahedra. In the t_2g_ and e_g_ states of an ML_6_ octahedron (Figure 3a,b), the d-orbitals of M make σ and π antibonding combinations with the p-orbitals of the ligands L. Thus, the p-orbital tails of the t_2g_ and e_g_ states are represented as in Figure 4a,b, respectively, so that each M-L bond has the p_π_ and p_σ_ tails in the t_2g_ and e_g_ states, respectively, as depicted in Figure 4c. The triple-degeneracy of the t_2g_ and the double-degeneracy of the e_g_ states are lifted in a ML_5_ square pyramid and a ML_4_ square plane, both of which have a four-fold rotational symmetry; the t_2g_ states (xz, yz, xy) are split into (xz, yz) and xy, and the e_g_ states (3z^2^ − r^2^, x^2^ − y^2^) into 3z^2^ − r^2^ and x^2^ − y^2^. Nevertheless, the description of the ligand p-orbital tails of the d-states depicted in Figure 4c remains valid.

### 2.3. Spin Exchanges in Terms of the p-Orbital Tails

In this section, we generalize the qualitative rules of spin exchanges formulated for the magnetic solids of Cu^2+^ ions [4]. Each Cu^2+^ ion has only one magnetic orbital, i.e., the x^2^−y^2^ state in which each Cu-L bond has a p_σ_ tail. The d-electron configuration of the magnetic ion is (t_2g_↑)^3^(e_g_↑)^2^(t_2g_↓)^0^(e_g_↓)^0^ in MnPS_3_, (t_2g_↑)^3^(e_g_↑)^2^(t_2g_↓)^1^(e_g_↓)^0^ in FePS_3_, (t_2g_↑)^3^(e_g_↑)^2^(t_2g_↓)^2^(e_g_↓)^0^ in CoPS_3_, and (t_2g_↑)^3^(e_g_↑)^2^(t_2g_↓)^3^(e_g_↓)^0^ in NiPS_3_. Thus, the Mn^2+^, Fe^2+^, Co^2+^, and Ni^2+^ ions possess 5, 4, 3, and 2 magnetic orbitals, respectively. For magnetic ions with several magnetic orbitals, the spin exchange J_AB_ between two such ions located at sites A and B is given by the sum of all possible individual exchanges $\left(\varphi_{i},\varphi_{j}\right)$,

(4) $$J_{AB}=\frac{2}{n_{A}n_{B}}\sum_{i\in A}\sum_{j\in B}(\varphi_{i},\varphi_{j})\propto \sum_{i\in A}\sum_{j\in B}(\varphi_{i},\varphi_{j})$$

where $n_{A}$ and $n_{B}$ are the number of magnetic orbitals at the sites A and B, respectively. Each individual exchange $\left(\varphi_{i},\varphi_{j}\right)$ can be FM or AFM depending on which term, J_F_ or J_AF_, dominates. Whether J_AB_ is FM or AFM depends on the sum of all individual $\left(\varphi_{i},\varphi_{j}\right)$ contributions.

#### 2.3.1. M-L-M Exchange

**Figure 5:**
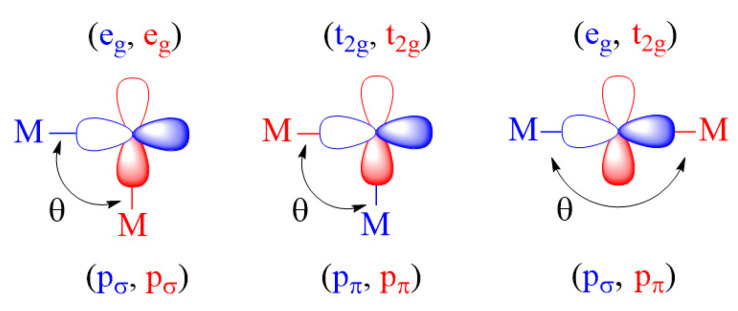
Three-types of M-L-M spin exchanges between t_2g_ and e_g_ magnetic orbitals.

As shown in Figure 5, there occur three types of M-L-M exchanges between the magnetic orbitals of t_2g_ and e_g_ states.

If the M-L-M bond angle θ is 90° for the (e_g_, e_g_) and (t_2g_, t_2g_) exchanges, and also when θ is 180° for the (e_g_, t_2g_) exchange, the two p-orbital tails have an orthogonal arrangement so that 〈$\varphi_{i}$|$\varphi_{j}$〉 = 0 (i.e., $J_{AF}$ = 0). However, the overlap electron density $\varphi_{i}\varphi_{j}$ is nonzero (i.e., $J_{F}$ ≠ 0), hence predicting these spin exchanges to be FM. When the θ angles of the (e_g_, e_g_) and (t_2g_, t_2g_) exchanges increase from 90° toward 180°, and also when the angle θ of the (e_g_, t_2g_) exchange decreases from 180° toward 90°, both $J_{AF}$ and $J_{F}$ are nonzero so that the balance between the two determines if the overall exchange $\left(\varphi_{i},\varphi_{j}\right)$ becomes FM or AFM. These trends are what the Goodenough–Kanamori rules [5,6,7,8] predict.

#### 2.3.2. M-L…L-M Exchange

**Figure 6:**
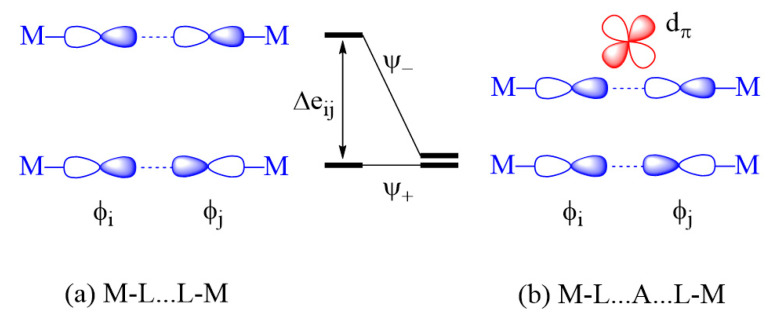
Interactions between the magnetic orbitals in the M-L…L-M exchange where their p_σ_ tails are pointing to each other. The large energy split $\Delta e_{ij}$ of the M-L…L-M exchange in (**a**) is reduced by the d_π_ orbital of the d^0^ cation A in the M-L…A…L-M exchange in (**b**).

**Figure 7:**
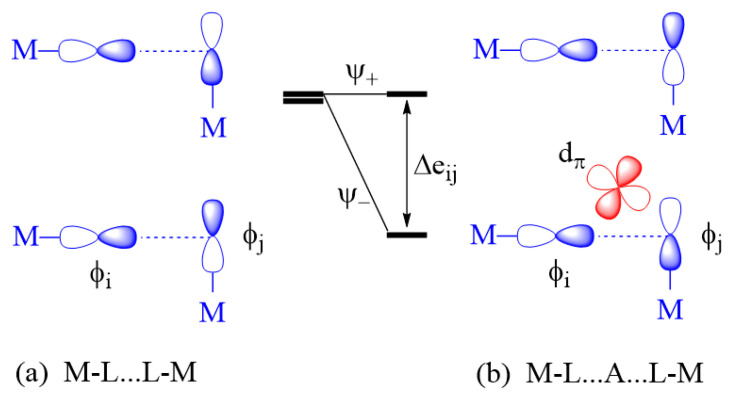
Interactions between the magnetic orbitals in the M-L…L-M exchange where their p_σ_ tails have an orthogonal arrangement. The small energy split $\Delta e_{ij}$ of the M-L…L-M exchange in (**a**) is enlarged by the d_π_ orbital of the d^0^ cation A in the M-L…A…L-M exchange in (**b**).

There are two extreme cases of M-L…L-M exchange. When the p_σ_-orbital tails are pointing toward each other (Figure 6a), the overlap integral, 〈$\varphi_{i}$|$\varphi_{j}$〉, can be substantial if the contact distance L…L lies in the vicinity of the van der Waals distance. However, the overlap electron density $\rho_{ij}=\varphi_{i}\varphi_{j}$ is practically zero because $\varphi_{i}$ and $\varphi_{j}$ do not have an overlapping region. Consequently, the in-phase and out-of-phase states Ψ_+_ and Ψ_-_ are split in energy with a large separation $\Delta e_{ij}$. Thus, it is predicted that the M-L…L-M type exchange can only be AFM [1,2,3,4]. When the L…L linkage is bridged by a d^0^ cation such as V^5+^ or W^6+^, for example, only the out-of-phase state Ψ_-_ is lowered in energy by the d_π_ orbital of the cation A, reducing the $\Delta e_{ij}$ so that the M-L…A…L-M exchange becomes weak (Figure 6b). Conversely, when the p-orbital tails of the M-L…L-M exchange path have an orthogonal arrangement (Figure 7a), the overlap integral, 〈$\varphi_{i}$|$\varphi_{j}$〉, is zero, making the M-L…L-M exchange weak. If the L…L linkage of such an exchange path is bridged by a d^0^ cation, the out-of-phase state Ψ_-_ level is lowered in energy enlarging $\Delta e_{ij}$ so that the M-L…A…L-M becomes strongly AFM (Figure 7b) [2,3,4].

In the M-L…A…L-M exchange of Figure 7, the vanishingly small $\Delta e_{ij}$ of the M-L…L-M exchange results because the two p_σ_ tails have an orthogonal arrangement. A very small $\Delta e_{ij}$ for the M-L…L-M exchange occurs even if the two M-L bonds are pointing to each other as in Figure 6 when one M-L bond has a p_σ_ tail and the other has a p_π_ tail, and also when both M-L bonds have p_π_ tails. Such M-L…L-M spin exchanges become strong in the corresponding M-L…A…L-M exchanges.

#### 2.3.3. Qualitative Rules Governing Spin Exchanges

The above discussions are based on the observation that the nature of a spin exchange, be it the M-L-M, M-L…L-M or M-L…A…L-M type, is governed by the ligand p-orbital tails present in the magnetic orbitals of the spin exchange path. The essential points of our discussions can be summarized as follows:

- For an individual $\left(\varphi_{i},\varphi_{j}\right)$ exchange of a M-L-M type, the (t_2g_, t_2g_) and (e_g_, e_g_) exchanges are FM if the bond angle θ is 90°, and so is the (t_2g_, e_g_) exchange if the bond angle θ is 180°. These exchanges become AFM when the bond angles θ deviate considerably from these values.
- An individual $\left(\varphi_{i},\varphi_{j}\right)$ exchange of a M-L…L-M or M-L…A…L-M type can only be AFM if not weak.
- A strong individual $\left(\varphi_{i},\varphi_{j}\right)$ exchange of a M-L…L-M is weakened by the d^0^ metal cation A in the M-L…A…L-M exchange, but a weak individual $\left(\varphi_{i},\varphi_{j}\right)$ exchange of a M-L…L-M is strengthened by the presence of a d^0^ metal cation A in the M-L…A…L-M exchange.
- When a magnetic ion has several unpaired spins, the spin exchange between two magnetic ions is given by the sum of all possible individual $\left(\varphi_{i},\varphi_{j}\right)$ exchanges.

These qualitative rules governing spin exchanges can serve as guidelines for exploring how the calculated spin exchanges are related to the structures of the exchange paths and also for ensuring that important exchange paths are included the set of spin exchanges to evaluate by the energy-mapping analysis.

## 3. Results and Discussion

### 3.1. Details of Calculations

We performed spin-polarized DFT calculations using the Vienna ab initio Simulation Package (VASP) [26,27], the projector augmented wave (PAW) method, and the PBE exchange-correlation functionals [28]. The electron correlation associated with the 3d states of M (M = Mn, Fe, Co, Ni) was taken into consideration by performing the DFT+U calculations [29] with the effective on-site repulsion U_eff_ = *U* − *J* = 4 eV on the magnetic ions. Our DFT + U calculations carried out for numerous magnetic solids of transition-metal ions showed that use of the U_eff_ values in the range of 3 − 5 eV correctly reproduce their magnetic properties (see the original papers cited in the review articles [1,2,3,22,24]). The primary purpose of using DFT + U calculations is to produce magnetic insulating states for magnetic solids. Use of U_eff_ = 3 − 5 eV in DFT + U calculations leads to magnetic insulating states for magnetic solids of Mn^2+^, Fe^2+^, Co^2+^, and Ni^2+^ ions. The present work employed the representative U_eff_ value of 4 eV. We carried out DFT + U calculations (with U_eff_ = 4 eV) to optimize the structures of MPS_3_ (M = Mn, Fe, Co, Ni) in their FM states by relaxing only the ion positions while keeping the cell parameters fixed and using a set of (4 × 2 × 6) k-points and the criterion of 5 × 10^−3^ eV/Å for the ionic relaxation. All our DFT + U calculations for extracting the spin-exchange parameters employed a (2*a*, 2*b*, *c*) supercell, the plane wave cutoff energy of 450 eV, the threshold of 10^−6^ eV for self-consistent-field energy convergence, and a set of (4 × 2 × 6) k-points. The preferred spin direction of the cation M^2+^ (M = Mn, Fe, Co, Ni) cation was determined by DFT + U + SOC calculations [30], employing a set of (4 × 2 × 6) k-points and the threshold of 10^−6^ eV for self-consistent-field energy convergence.

### 3.2. Magnetic Ground States of MPS_3_

We probed the magnetic ground states of the MPS_3_ phases by evaluating the relative energies, on the basis of DFT + U calculations, of the AF1, AF2 and AF3 spin configurations shown in Figure 2 as well as the FM, AF4, AF5, and AF6 states depicted in Supplementary Materials Figure S1. As summarized in Table 1, our calculations using the experimental structures of MPS_3_ show that the magnetic ground states of MnPS_3_ and NiPS_3_ adopt the honeycomb state AF1 and the ||*a*-chain state AF2, respectively, in agreement with experiment. In disagreement with experiment, however, the magnetic ground state is predicted to be the ||(*a* + *b*)-chain state AF3 for FePS_3_, and the honeycomb state AF1 for CoPS_3_. Since the energy differences between different spin ordered states are small, it is reasonable to speculate if they may be affected by small structural (monoclinic) distortion. Thus, we optimize the crystal structures of MPS_3_ (M = Mn, Fe, Co, Ni) by performing DFT + U calculations to obtain the structures presented in the supporting material. Then, we redetermined the relative stabilities of the FM and AF1–AF6 states using these optimized structures. Results of these calculations are also summarized in Table 1. The optimized structures predict that the magnetic ground states of MnPS_3_, CoPS_3_ and NiPS_3_ are the same as those observed experimentally, but that of FePS_3_ is still the ||(*a*+*b*)-chain state AF3 rather than the ||*a*-chain state AF2 reported experimentally. This result is not a consequence of using the specific value of U_eff_ = 4 eV, because our DFT + U calculations for FePS_3_ with U_eff_ = 3.5 and 4.5 eV lead to the same conclusion.

To resolve the discrepancy between theory and experiment on the magnetic ground state of FePS_3_, we note that the magnetic peak positions in the neutron diffraction profiles are determined by the repeat distances of the rectangular magnetic structures, namely, *a* and *b* for the AF2 state (Figure 2b), and *a*’ and *b*’ for the AF3 state (Figure 2c). In both the experimental and the optimized structures of FePS_3_, it was found that *a* = *a*’ = 5.947 Å and *b* = *b*’ = 10.300 Å. Thus, for the neutron diffraction refinement of the magnetic structure for FePS_3_, the AF2 and AF3 states provide an equally good model. In view of our computational results, we conclude that the AF3 state is the correct magnetic ground state for FePS_3_.

**Figure 8:**
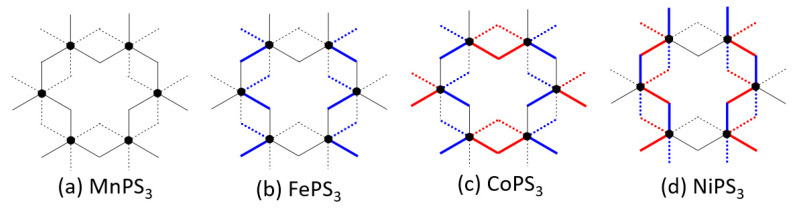
The arrangements of the M-S bond lengths of the MS_6_ octahedra in MPS_3_. The short M-S bonds are represented by blue lines, the medium M-S bonds by red lines, and the long M-S bonds by black lines.

The experimental and optimized structures of MPS_3_ (M = Mn, Fe, Co, Ni) are very similar, as expected. The important differences between them affecting the magnetic ground state would be the M-S distances of the MS_6_ octahedra, because the d-state splitting of the MS_6_ octahedra is sensitively affected by them. The M-S distances of the MS_6_ octahedra taken from the experimental and optimized crystal structures of MPS_3_ are summarized in Table 2, and their arrangements in the honeycomb layer are schematically presented in Figure 8. All Mn-S bonds of MnS_6_ in MnPS_3_ are nearly equal in length, as expected for a high-spin d^5^ ion (Mn^2+^) environment. The Fe-S bonds of FeS_6_ in the optimized structure of FePS_3_ are grouped into two short and four long Fe-S bonds. This distinction is less clear in the experimental structure. The Co-S bonds of CoS_6_ in the experimental and optimized structures of CoPS_3_ are grouped into two short, two medium and two long Co-S bonds. However, the sequence of the medium and long Co-S bonds is switched between the two structures. In the experimental and optimized structures of NiPS_3_, the Ni-S bonds of NiS_6_ are grouped into two short, two medium and two long Ni-S bonds. This distinction is less clear in the experimental structure. Thus, between the experimental and optimized structures of MPS_3_, the sequence of the two short, two medium and two long M-S bonds do not switch for M = Fe and Ni whereas it does for M = Co. The latter might be the cause for why the relative stabilities of the AF1 and AF2 states in CoPS_3_ switches between the experimental and optimized structures.

### 3.3. Preferred Spin Orientation of MPS_3_

#### 3.3.1. Quantitative Evaluation

We determine the preferred spin orientations of the M^2+^ ions in MPS_3_ (M = Mn, Fe, Co, Ni) phases by performing DFT + U + SOC calculations using their FM states with the ||z and ⊥z spin orientations. For the ⊥z direction we selected the ||a-direction. As summarized in Table 3, these calculations predict the preferred spin orientation to be the ||z direction for FePS_3_, and the ||x direction for MnPS_3_, CoPS_3_ and NiPS_3_. These predictions are in agreement with experiment for FePS_3_ [9,18], CoPS_3_ [19], and NiPS_3_ [20], while this is not the case for MnPS_3_ [9,12,14,31]. Our DFT + U + SOC calculations for the AF1 state of MnPS_3_ show that the ||x spin orientation is still favored over the ||z orientation just as found from the calculations using the FM state of MnPS_3_. The Mn^2+^ spins of MnPS_3_ were reported to have the ||z orientation in the early studies [9,12], but were found to be slightly tilted away from the z-axis (by 8°) [14,31]. In our further discussion (see below), this small deviation is neglected.

#### 3.3.2. Qualitative Picture

##### Selection Rules of Spin Orientation and Implications

With the local z-axis of a ML_6_ octahedron along its three-fold rotational axis (Figure 1a), the t_2g_ set is described by {1a, 1e’}, and the e_g_ set by {2e’}[22,23,24], where

(5) $$\begin{array}{l} 1a=3z^{2}-r^{2}\\ \left\{1e^{\prime }\right\}=\left\{\sqrt{\frac{2}{3}}xy-\sqrt{\frac{1}{3}}xz,\sqrt{\frac{2}{3}}(x^{2}-y^{2})-\sqrt{\frac{1}{3}}yz\right\}\\ \left\{2e^{\prime }\right\}=\left\{\sqrt{\frac{1}{3}}xy+\sqrt{\frac{2}{3}}xz,\sqrt{\frac{1}{3}}(x^{2}-y^{2})+\sqrt{\frac{2}{3}}yz\right\} \end{array}$$

**Figure 9:**
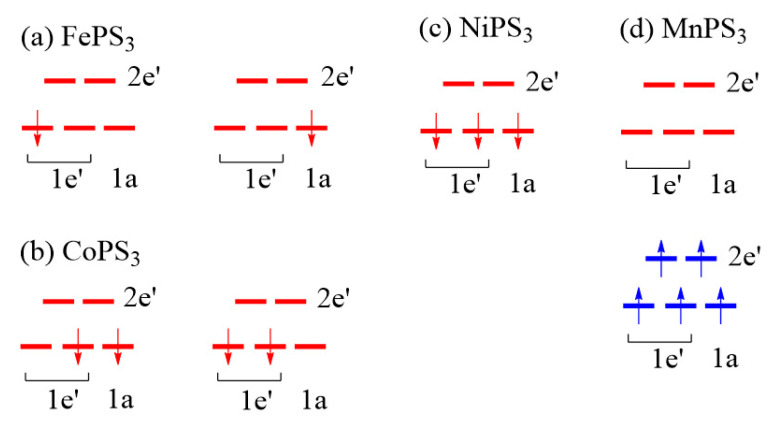
Electron configurations of the M^2+^ (M = Mn, Fe, Co, Ni) ions of (**a**) FePS_3_, (**b**) CoPS_3_, (**c**) NiPS_3_, and (**d**) MnPS_3_ in the spin polarized description. In (**a**–**c**), the up-spin d-states lying below the down-spin t_2g_ states are not shown for clarity.

Using these d-states, the electron configurations expected for the M^2+^ ions of MPS_3_ (M = Mn, Fe, Co, Ni) are presented in Figure 9. In the spin polarized description of a magnetic ion, the up-spin d-states lie lower in energy than the down-spin states so that the HOMO and LUMO occur in the down-spin d-states for the M^2+^ ions with more than the d^5^ electron count, so only the down-spin states are shown for FePS_3_, CoPS_3_, and NiPS_3_ in Figure 9a–c. For MnPS_3_ with d^5^ Mn^2+^ ion, the HOMO is represented by the up-spin 1e’, and the LUMO by the down-spin 1a and 2e’ (Figure 9d).

In terms of the d-orbital angular states |*L*, *L*_z_〉 (*L* = 2, *L*_z_ = −2, −1, 0, 1, 2), the 1e’ state consists of the |2, ±2〉 and |2, ±1〉 sets in the weight ratio of 2:1, and the 2e’ state in the weight ratio of 1:2 ratio. Consequently, the major component of the 1e’ set is the |2, ±2〉 set, while that of the 2e’ set is the |2, ±1〉 set.

The selection rules of the spin orientation are based on the |Δ*L*_z_| value between the HOMO and LUMO of ML_n_. If the HOMO and LUMO both occur in the up-spin state or in down-spin states (Figure 9a–c), the ||z spin orientation is predicted if |Δ*L*_z_| = 0, and the ⊥z spin orientation if |Δ*L*_z_| = 1. When |Δ*L*_z_| > 1, the HOMO and LUMO do not interact under SOC and hence do not affect the spin orientation. Between the 1a, 1e’ and 2e’ states, we note the following cases of values:

(6) $$\left|\Delta L_{z}\right|=0\{\begin{array}{c} \mathrm{between}\;\mathrm{the}\;\mathrm{major}\;\mathrm{components}\;\mathrm{of}\;\mathrm{the}\;1e^{\prime }\;\mathrm{set}\\ \mathrm{between}\;\mathrm{the}\;\mathrm{major}\;\mathrm{components}\;\mathrm{of}\;\mathrm{the}\;2e^{\prime }\;\mathrm{set} \end{array}$$

(7) $$\left|\Delta L_{z}\right|=1\{\begin{array}{c} \mathrm{between}\;1a\;\mathrm{and}\;\mathrm{the}\;\mathrm{minor}\;\mathrm{component}\;\mathrm{of}\;1e^{\prime }\\ \mathrm{between}\;1a\;\mathrm{and}\;\mathrm{the}\;\mathrm{major}\;\mathrm{component}\;\mathrm{of}\;2e^{\prime }\\ \mathrm{between}\;\mathrm{the}\;\mathrm{major}\;\mathrm{components}\;\mathrm{of}\;1e^{\prime }\;\mathrm{and}\;2e^{\prime } \end{array}$$

We now examine the preferred spin orientations of MPS_3_ from the viewpoint of the selection rules and their electron configurations (Figure 9). The d-electron configuration of FePS_3_ can be either (d↑)^5^(1e’↓)^1^ or (d↑)^5^(1a↓)^1^ (Figure 9a), where the notation (d↑)^5^ indicates that all up-spin d-states are occupied. The (d↑)^5^(1e’↓)^1^ configuration, for which |Δ*L*_z_| = 0, predicts the ||z spin orientation, while the (d↑)^5^(1a↓)^1^ configuration, for which |Δ*L*_z_| = 1, predicts the ⊥z spin orientation. Thus, the (d↑)^5^(1a↓)^1^ configuration is correct for the Fe^2+^ ion of FePS_3_. Since this configuration has the degenerate level 1e’ unevenly occupied, it should possess uniaxial magnetism [2,3,22,23,24] and hence a large magnetic anisotropy energy. This is in support of the experimental finding of the Ising character of the spin lattice of FePS_3_ [16] or the single-ion anisotropic character of the Fe^2+^ ion [17,18]. The d-electron configuration of CoPS_3_ can be either (d↑)^5^(1e’↓)^2^ or (d↑)^5^(1a↓)^1^(1e’↓)^1^ (Figure 9b). The (d↑)^5^(1e’↓)^2^ configuration, for which |Δ*L*_z_| = 1, predicts the ⊥z spin orientation, while the (d↑)^5^(1a↓)^1^(1e’↓)^1^ configuration, for which |Δ*L*_z_| = 0, predicts the ||z spin orientation. Thus, the (d↑)^5^(1e’↓)^2^ configuration is correct for the Co^2+^ ion of CoPS_3_. Since this configuration has the degenerate level 1e’ evenly occupied, it does not possess uniaxial magnetism [2,3,22,23,24] and hence a small magnetic anisotropy energy. The d-electron configuration of NiPS_3_ is given by (d↑)^5^(1a)^1^(1e’↓)^2^ (Figure 9c), for which |Δ*L*_z_| = 1, so the ⊥z spin orientation is predicted in agreement with experiment.

Let us now consider the spin orientation of the Mn^2+^ ion of MnPS_3_. First, it should be noted that, if the HOMO and LUMO occur in different spin states as in MnPS_3_ (Figure 9d), the selection rules predict the opposite to those found for the case when the HOMO and LUMO occur all in up-spin states or all in down-spin states [2,3,22,23,24]. Namely, the preferred spin orientation is the ||z spin orientation if |Δ*L*_z_| = 1, but the ⊥z spin orientation if |Δ*L*_z_| = 0 [2,3,22,23,24]. According to Equation (7), |Δ*L*_z_| = 1 for the Mn^2+^ ion of MnPS_3_, which predicts the ⊥z orientation as the preferred spin direction in agreement with the quantitative estimate of the magnetic anisotropy energy obtained from the DFT + U + SOC calculations, although this is in disagreement with experiment [5,8,9,10]. It has been suggested that the ||z spin orientation is caused by the magnetic dipole–dipole (MDD) interactions [13]. This subject will be probed in the following.

##### Magnetic Dipole–Dipole Interactions

Being of the order of 0.01 meV for two spin-1/2 ions separated by 2 Å, the MDD interaction is generally weak. For two spins located at sites i and j with the distance r_ij_ and the unit vector e_ij_ along the distance, the MDD interaction is defined as [32]

(8) $$\left(\frac{g^{2}\mu_{B}^{2}}{a_{0}^{3}}\right){\left(\frac{a_{0}}{r_{ij}}\right)}^{3}\left[-3({\vec{S}}_{i}\cdot {\vec{e}}_{ij})({\vec{S}}_{j}\cdot {\vec{e}}_{ij})\;+({\vec{S}}_{i}\cdot {\vec{S}}_{j})\right]$$

where a_0_ is the Bohr radius (0.529177 Å), and (gμ_B_)^2^/(a_0_)^3^ = 0.725 meV. The MDD effect on the preferred spin orientation of a given magnetic solid can be examined by comparing the MDD interaction energies calculated for a number of ordered spin arrangements. In summing the MDD interactions between various pairs of spin sites, it is necessary to employ the Ewald summation method [33,34,35]. Table 4 summarizes the MDD interaction energies calculated, by using the optimized structures of MPS_3_ (M = Mn, Fe, Co, Ni), for the ||z and ||x spin directions in the AF1, AF2 and AF3 states. The corresponding results obtained by using the experimental structures of MPS_3_ are summarized in Table S1.

These results can be summarized as follows: for the ||z spin orientation, the AF1 state is more stable than the AF2 and AF3 states. For the ||x spin orientation, the AF2 state is more stable than the AF1 and AF3 states. The ||x spin direction of the AF2 state is more stable than the ||z spin direction of the AF1 state. However, none of these results can reverse the relative stabilities of the ||z and ||x spin directions determined for FePS_3_, CoPS_3_, and NiPS_3_ from the DFT + U + SOC calculations (Table 3). The situation is slightly different for MnPS_3_, which adopts the AF1 state as the magnetic ground state. For MnPS_3_ in this state, the MDD calculations predict that the ||z spin orientation is more stable than the ||x spin orientation by 0.3 K per formula unit (Table 4). Note that this prediction is the exact opposite to what the DFT + U + SOC calculations predict for MnPS_3_ in the AF1 state (Table 3). Thus, the balance between these two opposing energy contributions will determine whether the ||z spin orientation is more stable than the ⊥z spin orientation in agreement with the experimental observation. Consequently, for MnPS_3_ the MDD interaction dominates over the SOC effect which is plausible because of the half-filled shell electronic configuration. This is because the AF1 magnetic structure is forced on MnPS_3_; in terms of purely MDD interactions alone, the ⊥z spin orientation in the AF2 state is most stable.

### 3.4. Quantitative Evaluations of Spin Exchanges

**Figure 10:**
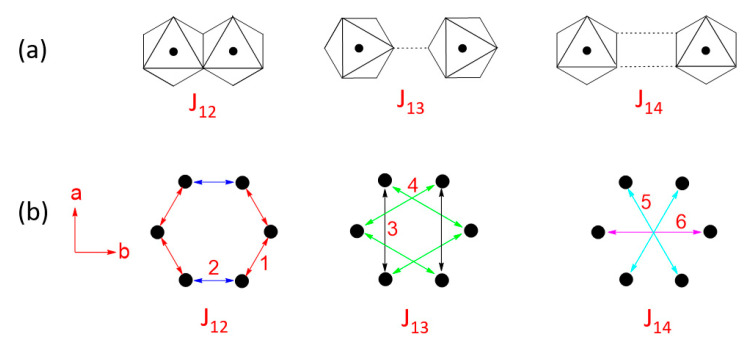
(**a**) Three kinds of spin exchange paths in each honeycomb layer of MPS_3_. (**b**) Two kinds of the spin exchanges resulting from J_12_, J_13_ and J_14_ due to the loss of the trigonal symmetry in the MPS_3_ honeycomb layers. In (**b**), the numbers 1–6 refer to J_1_–J_6_, respectively.

Due to the monoclinic crystal structure that MPS_3_ adopts, each of the exchanges J_12_, J_13_ and J_14_ (Figure 10a) are expected to split into two slightly different spin exchanges (Figure 10b) so that there are six spin exchanges J_1_–J_6_ to consider. To extract the values of the six spin exchanges J_1_–J_6_ (Figure 3), we employ the spin Hamiltonian expressed as:

(9) $$H_{spin}=-\sum_{i>j}J_{ij}{\hat{S}}_{i}\cdot {\hat{S}}_{j}$$

Then, the energies of the FM and AF1–AF6 states of MPS_3_ (M = Mn, Fe, Co, Ni) per 2 × 2 × 1 supercell are written as:

E_FM_ = (–16J_1_ − 8J_2_ − 16J_3_ − 32J_4_ − 16J_5_ − 8J_6_)S^2^
     E_AF1_ = (+16J_1_ + 8J_2_ − 16J_3_ − 32J_4_ + 16J_5_ + 8J_6_)S^2^
     E_AF2_ = (−16J_1_ + 8J_2_ − 16J_3_ + 32J_4_ + 16J_5_ + 8J_6_)S^2^
E_AF3_ = (−8J_2_ + 16J_3_ + 16J_5_ + 8J_6_)S^2^      
     E_AF4_ = (+16J_1_ − 8J_2_ − 16J_3_ + 32J_4_ − 16J_5_ − 8J_6_)S^2^
E_AF5_ = (+8J_2_ + 16J_3_ − 16J_5_ + 8J_6_)S^2^      
E_AF6_ = (−8J_2_ + 16J_3_ + 16J_5_ − 8J_6_)S^2^      

where S is the spin on each M^2+^ ion (i.e., S = 5/2, 2, 3/2 and 1 for M = Mn, Fe, Co, and Ni, respectively). By mapping the relative energies of the FM and AF1–AF6 states determined in terms of the spin exchange J_1_–J_6_ onto the corresponding relative energies obtained from the DFT + U calculations (Table 1), we find the values of J_1_–J_6_ listed in Table 5. (The spin exchanges of MPS_3_ determined by using their experimental crystal structures are summarized in Table S2)

With the sign convention adopted in Eq. 1, AFM exchanges are represented by J_ij_ < 0, and FM exchanges by J_ij_ > 0. From Table 5, the following can be observed:

- In all MPS_3_ (M = Mn, Fe, Co, Ni), J_1_ ≠ J_2_, J_3_ ≠ J_4_, and J_5_ ≠ J_6_, reflecting that the exchange paths are different between J_1_ and J_2_, between J_3_ and J_4_, and between J_5_ and J_6_ (Figure 10).
- J_1_ ≈ J_2_ < 0, J_3_ ≈ J_4_ ≈ 0, and J_5_ ≈ J_6_ < 0 for MnPS_3_ while J_1_ ≈ J_2_ > 0, J_3_ ≈ J_4_ ≈ 0, and J_5_ ≈ J_6_ < 0 NiPS_3_. To a first approximation, the electron configurations of MnPS_3_ and NiPS_3_ can be described by (t_2g_)^3^(e_g_)^2^ and (t_2g_)^6^(e_g_)^2^, respectively. That is, they do not possess an unevenly occupied degenerate state t_2g_.
- In FePS_3_ and CoPS_3_, J_1_ and J_2_ are quite different, and so are J_3_ and J_4_. While J_5_ and J_6_ are comparable in FePS_3_, they are quite different in CoPS_3_. The electron configurations of FePS_3_ and NiPS_3_ can be approximated by (t_2g_)^4^(e_g_)^2^ and (t_2g_)^5^(e_g_)^2^, respectively. Namely, they possess an unevenly occupied degenerate state t_2g_.
- The strongest exchange is J_1_ in MnPS_3_, but J_6_ in other MPS_3_ (M = Fe, Co, Ni).
- The second NN exchange J_3_ is strongly FM in CoPS_3_, while the third NN exchange J_6_ is very strongly AFM in CoPS_3_ and NiPS_3_.

From the viewpoints of the expected trends in spin exchanges, the observation (e) is quite unusual. This will be discussed in the next section.

### 3.5. Unusual Features of the M-L…L-M Spin Exchanges

#### 3.5.1. Second Nearest-Neighbor Exchange

**Figure 11:**
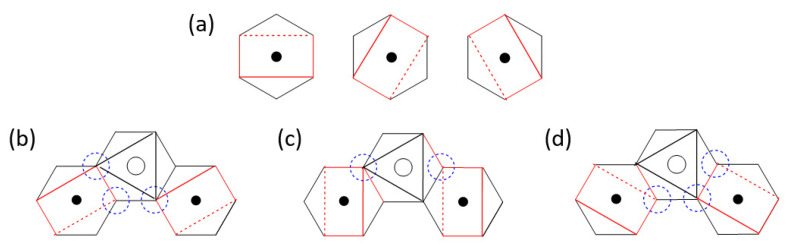
(**a**) Three MS_4_ square planes of a MS_6_ octahedron, containing the xy, xz and yz states of an MS_6_ octahedron. (**b**–**d**) Three cases of the CoS_4_ square planes containing the t_2g_ magnetic orbitals in the J_3_ exchange path of CoPS_3_.

As pointed out in the previous section, the second NN exchange J_3_ of CoPS_3_ is strongly FM despite that it is a M-L…L-M exchange to a first approximation. This implies that the J_F_ component in some $\left(\varphi_{i},\varphi_{j}\right)$ exchanges is nonzero, namely, the overlap electron density associated with those exchanges is nonzero. This implies that the p-orbital tails of the two magnetic orbitals are hybridized with the group orbitals of the P_2_S_6_^4−^ anion, i.e., they become delocalized into the whole P_2_S_6_^4−^ anion. Each MS_6_ octahedron has three mutually orthogonal “MS_4_ square planes” containing the yz, xz and xy states (Figure 11a). At the four corners of these three square planes, the p-orbital tails of the d-states are present (Figure 3a).

The lone-pair orbitals of the S atoms are important for the formation of each MS_6_ octahedron. Due to the bonding requirement of the P_2_S_6_^4−^ anion, such lone pair orbitals become symmetry-adapted. An example in which the p-orbitals of all the S atoms are present is shown in Figure 1e.

With the (t_2g_)^5^(e_g_)^2^ configuration, the Co^2+^ ion of CoPS_3_ has five electrons in the t_2g_ level, namely, it has only one t_2g_ magnetic orbital. This magnetic orbital is contained in one of the three CoS_4_ square planes presented in Figure 11b–d. When the S p-orbital at one corner of the P_2_S_6_^4−^ anion interacts with a d-orbital of M, the S p-orbitals at the remaining corners are also mixed in. Thus, when P_2_S_6_^4−^ anion shares corners with both MS_4_ square planes of the J_3_ exchange path, a nonzero overlap electron density is generated, thereby making the spin exchange FM. For convenience, we assume that the magnetic t_2g_ orbital of the Co^2+^ ion is the xy state. Then, there will be not only the (xy, xy) exchange, but also the (xy, x^2^−y^2^) and (x^2^−y^2^, xy) exchanges between the two Co^2+^ ions of the J_3_ path. All these individual exchanges lead to nonzero overlap electron densities by the delocalization of the p-orbital tails with the group orbitals of the molecular anion P_2_S_6_^4−^. In other words, the spin exchange J_3_ in CoPS_3_ is nominally a M-L…L-M, which is expected to be AFM by the qualitative rule, but it is strongly FM. It is clear that, if the L…L linkage is a part of the covalent framework of a molecular anion such as P_2_S_6_^4−^, a nominal M-L…L-M exchange can become FM for a certain combination of the d-electron count of the metal M and the geometries of the exchange path.

#### 3.5.2. Third Nearest-Neighbor Exchange

**Figure 12:**
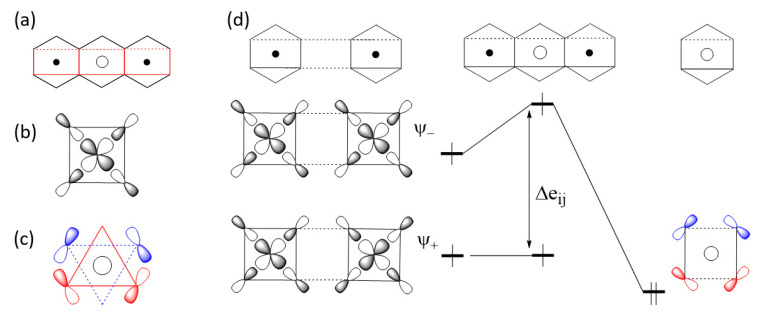
(**a**) The J_6_ spin exchange path of MPS_3_ (M = Co, Ni) viewed in terms of the MS_4_ and P_2_S_4_ square planes. (**b**) The x^2^-y^2^ magnetic orbital of the MS_6_ octahedron. (**c**) The S p-orbitals present at the corners of the P_2_S_4_ square plane. (**d**) How the M-S…S-M spin exchange is enhanced by the through-bond effect of the intervening P_2_S_6_ octahedron.

Unlike in MnPS_3_ and FePS_3_, the M-S…S-M exchange J_6_ is unusually strong in CoPS_3_ and NiPS_3_ (Section 3.3). This is so despite that the S…S contact distances are longer in CoPS_3_ and NiPS_3_ than in MnPS_3_ and FePS_3_ (i.e., the S…S contact distance of the J_6_ path in MPS_3_ is 3.409, 3.416, 3.421 and 3.450 Å for M = Mn, Fe, Co and Ni, respectively). We note that a strong M-L…L-M exchange (i.e., a spin exchange leading to a large energy split $\Delta e_{ij}$) becomes weak, when the L…L contact is bridged by a d^0^ cation like, e. g., V^5+^ and W^6+^ to form the M-L…A…L-M exchange path (Figure 6b), because the out-of-phase combination $\psi_{-}$ is lowered in energy by interacting with the unoccupied d_π_ orbital of the cation A. Conversely, then, one may ask if the strength of a M-L…L-M spin exchange can be enhanced by raising the $\psi_{-}$ level. The latter can be achieved if the L…L path provides an occupied level of π-symmetry that can interact with $\psi_{-}$. As depicted in Figure 12a, the J_6_ path has the two MS_4_ square planes containing the x^2^-y^2^ magnetic orbitals (Figure 12b). The lone-pair group orbital of the S_4_ rectangular plane (Figure 12c) of the P_2_S_6_^4−^ anion has the correct symmetry to interact with $\psi_{-}$, so that the $\psi_{-}$ level is raised in energy thereby enlarging the energy split between $\psi_{+}$ and $\psi_{-}$ and strengthening the J_6_ exchange (Figure 12d). Although this reasoning applies equally to MnPS_3_ and FePS_3_, the latter do not have a strong J_6_ exchange. This can be understood by considering Equation (1), which shows that a magnetic ion with several magnetic orbitals leads to several individual spin exchanges that can lead to FM contributions.

In view of the above discussion, which highlights the unusual nature of the second and third NN spin exchanges mediated by a molecular anion such as P_2_S_6_^4−^, we propose to use the notation M-(L-L)-M to distinguish it from M-L-M. M-L…L-M and M-L…A…L-M type exchanges. The notation (L-L) indicates two different ligand sites of a multidentate molecular anion, each with lone pairs for the coordination with a cation M. Such M-(L-L)-M exchanges can be strongly FM or strongly AFM, as discussed above. Currently, there are no qualitative rules with which to predict whether they will be FM or AFM. A similar situation was found, for example, for the mineral Azurite Cu_3_(CO_3_)_2_(OH)_2_, in which every molecular anion CO_3_^2−^ participates in three different Cu-(O-O)-Cu exchanges. DFT + U calculations show that one of these three is substantially AFM, but the remaining two are negligible. So far, this observation has not been understood in terms of qualitative reasoning.

### 3.6. Description Using Three Exchanges

Experimentally, the magnetic properties of MPS_3_ have been interpreted in terms of three exchange parameters, namely, by assuming that J_1_ = J_2_ (≡ J_12_), J_3_ = J_4_ (≡ J_13_), and J_5_ = J_6_ (≡ J_14_). To investigate whether this simplified description is justified, we simulate the relative energies of the seven ordered spin states of MPS_3_ by using the three exchanges J_12_, J_13_ and J_14_ as parameters in terms of the least-square fitting analysis. Our results, summarized in Table 6, show that the standard deviations of J_12_, J_13_ and J_14_ are small for MnPS_3_ and NiPS_3_, moderate in FePS_3_, but extremely large in CoPS_3_ (for details, see Figures S2–S5). The exchanges experimentally deduced for FePS_3_ are J_12_ = −17 K, J_13_ = −0.5 K, and J_14_ = 7 K from neutron inelastic scattering measurements [17], −17 K ≤ J_12_ ≤ −5.6 K, −7.2 K ≤ J_13_ ≤ 2.8 K, and 0 ≤ J_14_ ≤ 10 K from powder susceptibility measurements [9], and J_12_ = −19.6 K, J_13_ = 10.3 K, and J_14_ = −2.2 K from high field measurements [17]. These experimental estimates are dominated by J_12_, but the theoretical estimates of Table 6 by J_14_. One might note from Table 6 that the magnetic properties of MnPS_3_, FePS_3_ and NiPS_3_ can be reasonably well approximated by two exchanges, that is, by J_12_ and J_14_ for MnPS_3_, by J_14_ and J_12_ for NiPS_3_, and by J_14_ and J_13_ for FePS_3_. However, this three-parameter description leads to erroneous predictions for the magnetic ground states of MPS_3_; it predicts the AF1 state to be the ground state for both MnPS_3_ and CoPS_3_. This prediction is correct for MnPS_3_, but incorrect for CoPS_3_. In addition, it predicts that the AF2 and AF3 states possess the same stability for all MPS_3_ (M = Mn, Fe, Co, Ni), and are the ground states for FePS_3_ and NiPS_3_. These two predictions are both incorrect.

## 4. Concluding Remarks

Our DFT + U calculations for the optimized structures of MPS_3_ (M = Mn, Fe, Co, Ni) reveal that, in agreement with experiment, the magnetic ground state of MnPS_3_ is the AF1 state while those of CoPS_3_ and NiPS_3_ are the AF2 state. In disagreement with experiment, however, our calculations predict the AF2 state to be the magnetic ground state for FePS_3_. Our DFT + U + SOC calculations show that, in agreement with experiment, the preferred spin orientation of FePS_3_ is the ||z direction while those of CoPS_3_ and NiPS_3_ are the ⊥z direction, and the Fe^2+^ ion of FePS_3_ exhibits uniaxial anisotropy. In disagreement with experiment, these calculations predict the preferred spin orientation for MnPS_3_ to be the ⊥z direction. Our analyses suggest that the ||z spin direction experimentally observed for the Mn^2+^ ions arises from the magnetic dipole–dipole interactions in the AF1 magnetic state. We presented simple qualitative rules governing spin exchanges to be used as guidelines for gauging the nature of various spin exchanges. These rules allowed us to recognize several unusual exchanges of MPS_3_; the second NN exchange J_3_ of CoPS_3_ is strongly FM while the third NN exchanges J_6_ of CoPS_3_ and NiPS_3_ are very strongly AFM. These observations reflect the fact that the lone-pair orbitals of the P_2_S_6_^4−^ ion, mediating the spin exchanges in MPS_3_ are symmetry-adapted group orbitals, so the effect of coordinating one S atom to one M^2+^ ion is felt by all the remaining five S atoms of P_2_S_6_^4−^. The spin exchanges mediated by molecular anions, termed the M-(L-L)-M type exchanges, differ in nature from the M-L-M, M-L…L-M and M-L…A…L-M type exchanges. To find qualitative trends in the M-(L-L)-M type exchanges, it is necessary to further study the spin exchanges involving various other molecular anions.

## Figures and Tables

**Table 1:** Relative energies (in meV/formula unit) obtained for the seven ordered spin states of MPS_3_ (M = Mn, Fe, Co, Ni) from DFT + U calculations with U_eff_ = 4 eV. The numbers without parentheses are obtained by using the experimental structures, and those in parentheses by using the structures optimized by DFT + U calculations.

	Mn	Fe	Co	Ni
FM	33.77 (33.36)	31.25 (25.10)	71.46 (55.00)	45.00 (42.04)
AF1	0 (0)	12.24 (5.16)	0 (5.70)	6.50 (7.11)
AF2	15.54 (15.50)	12.92 (7.93)	45.05 (0)	0 (0)
AF3	14.25 (14.21)	0 (0)	34.02 (24.99)	0.35 (0.34)
AF4	14.72 (14.45)	20.85 (18.57)	22.16 (26.00)	52.40 (49.53)
AF5	12.77 (12.58)	15.79 (12.95)	157.25 (158.33)	33.62 (31.98)
AF6	17.24 (17.07)	10.57 (6.33)	140.58 (143.05)	16.43 (15.21)

**Table 2:** The M-S bond distances (in Å) of the MS_6_ octahedra in MPS_3_ (M = Mn, Fe, Co, Ni) obtained from the experimental and the optimized crystal structures, which are shown without and with parentheses, respectively.

Mn	Fe	Co	Ni
2.627 (2.632)	2.546 (2.525)	2.485 (2.492)	2.457 (2.453)
2.627 (2.632)	2.546 (2.526)	2.485 (2.492)	2.457 (2.453)
2.625 (2.635)	2.547 (2.571)	2.504 (2.525)	2.462 (2.457)
2.625 (2.635)	2.547 (2.572)	2.504 (2.525)	2.462 (2.457)
2.634 (2.639)	2.549 (2.572)	2.491 (2.537)	2.465 (2.461)
2.634 (2.639)	2.549 (2.573)	2.491 (2.537)	2.465 (2.461)

**Table 3:** Relative energies (in K per formula unit) of the ||z and ⊥z spin orientations of the M^2+^ ions in the FM states of MPS_3_ (M = Mn, Fe, Co, Ni) obtained by DFT + U + SOC calculations. The results calculated by using the optimized (experimental) structures are presented without (with) the parentheses.

	MnPS_3_^a^	FePS_3_^b^	CoPS_3_	NiPS_3_
⊥z	0 (0)	20.0 (21.8)	0 (0)	0 (0)
||z	0.3 (0.3)	0 (0)	3.8 (5.2)	0.8 (0.7)

^a^ The same result is obtained by using the AF1 state, which is the magnetic ground state of MnPS_3_. ^b^ The same results are obtained from our DFT+U calculations with U_eff_ = 3.5 and 4.5 eV.

**Table 4:** Relative energies (in K per formula unit) of the ||x and ||z spin orientations calculated by MDD calculations for the M^2+^ ions of MPS_3_ (M = Mn, Fe, Co, Ni) in the AF1, AF2 and AF3 states using the optimized crystal structures.

	MnPS_3_		FePS_3_		CoPS_3_		NiPS_3_	
	||x	||z	||x	||z	||x	||z	||x	||z
AF1	0.48	0.17	0.36	0.12	0.21	0.07	0.09	0.03
AF2	0.00	0.35	0.00	0.26	0.00	0.15	0.00	0.07
AF3	0.55	0.38	0.38	0.27	0.22	0.15	0.10	0.07

**Table 5:** Spin exchanges J_1_–J_6_ obtained (for the optimized structures of MPS_3_ (M = Mn, Fe, Co, Ni) from DFT + U calculations with U_eff_ = 4 eV) by simulating the relative energies of the FM and AF1–AF6 states with the six spin exchanges.

	Mn	Fe	Co	Ni
J_1_	1.00	0.37	0.05	−0.25
J_2_	0.87	−0.32	−0.91	−0.14
J_3_	0.06	0.36	−0.55	0.04
J_4_	0.05	0.07	0.04	−0.01
J_5_	0.34	0.86	0.11	0.99
J_6_	0.33	1.00	1.00	1.00
	J_1_ = −16.0 K	J_6_ = −18.4 K	J_6_ = −608.7 K	J_6_ = −172.4 K

**Table 6:** Spin exchanges J_12_, J_13_ and J_14_ in K obtained (for the optimized structures of MPS_3_ (M = Mn, Fe, Co, Ni) from DFT + U calculations with U_eff_ = 4 eV) by simulating the relative energies of the FM and AF1–AF6 states with the three spin exchanges.

	Mn	Fe	Co	Ni
J_12_	−15.5 ± 0.4	2.0 ± 7.7	−61.4 ± 119.0	36.3 ± 4.3
J_13_	−0.9 ± 0.2	−7.7 ± 3.9	60.7 ± 55.3	0.0 ± 2.0
J_14_	−5.3 ± 0.3	−20.9 ± 4.5	−59.1 ± 95.6	−186.0 ± 3.4

## Supplementary Materials

The following are available online. Figure S1: Ordered spin states, FM, AF4, AF5 and AF6 employed together with the states AF1, AF2 and AF3 (see the text) to determine the magnetic ground states as well as the spin exchanges J_1_–J_6_ of MPS_3_ (M = Mn, Fe, Co, Ni). Figure S2–S5. Results of the least-square fitting the relative energies of the seven ordered spin states (FM, AF1-AF6) of MnPS_3_, CoPS_3_ and FePS_3_, and NiPS_3_, Table S1: Relative energies (in K per formula unit) of the ||x and ||z spin orientations obtained by MDD calculations for the M^2+^ ions of MPS_3_ (M = Mn, Fe, Co, Ni) in the AFM1, AF2 and AFM3 states using the experimental crystal structures. Table S2: Spin exchanges (in K) obtained for the experimental structures of MPS_3_ (M = Mn, Fe, Co, Ni) from DFT + U calculations with U_eff_ = 4 eV.
