# Supplementary material for: Unusual Spin Exchanges Mediated by the Molecular Anion P2S64−: Theoretical Analyses of the Magnetic Ground States, Magnetic Anisotropy and Spin Exchanges of MPS3 (M = Mn, Fe, Co, Ni)
Source: Molecules. 2021 Mar 5;26(5):1410. doi: 10.3390/molecules26051410 (PMC7961718; doi:10.3390/molecules26051410)
Supplement: Supplementary file 1 [file molecules-26-01410-s001.zip › Supplementary/molecules-1107020 suppmentary.pdf]

Supporting Information

for

Unusual spin exchanges mediated by the molecular anion $P_2S_6^{4-}$: Theoretical analyses of the magnetic ground states, magnetic anisotropy and spin exchanges of MPS_3 (M = Mn, Fe, Co, Ni)

Hyun-Joo Koo[a,*], Reinhard K. Kremer[b] and Myung-Hwan Whangbo[a,c,*]

[a] Department of Chemistry and Research Institute for Basic Sciences, Kyung Hee University, Seoul 02447, Republic of Korea

[b] Max Planck Institute for Solid State Research, Heisenbergstrasse 1, D-70569 Stuttgart, Germany

[c] Department of Chemistry, North Carolina State University, Raleigh, NC 27695-8204, USA

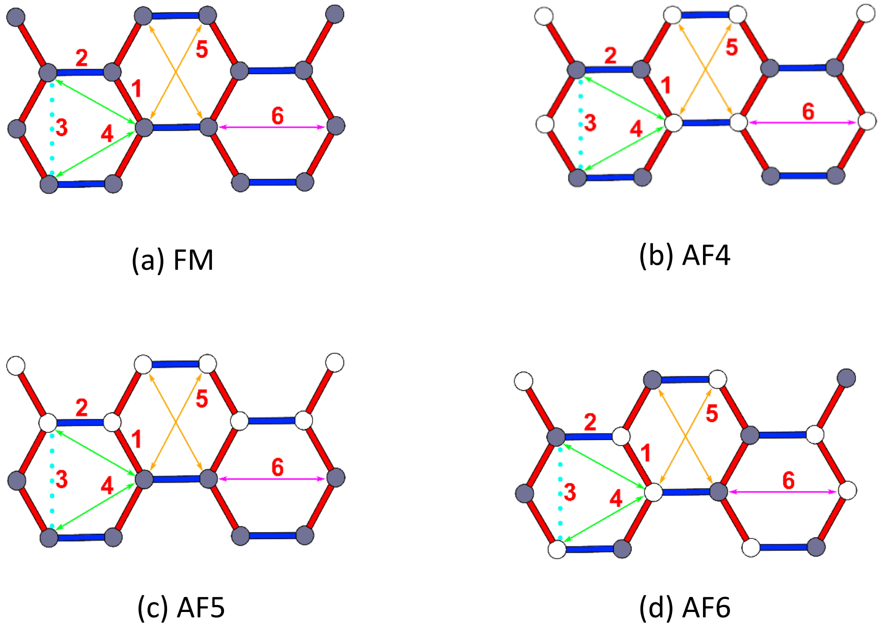

Figure S1. Ordered spin states, FM, AF4, AF5 and AF6 employed together with the states AF1, AF2 and AF3 (see the text) to determine the magnetic ground states as well as the spin exchanges J_1 – J_6 of MPS_3 (M = Mn, Fe, Co, Ni). Here the shades and unshaded circles reoresent the up- and down-spins, respectively, and the numbers 1 – 6 refer to J_1 – J_6, respectively.

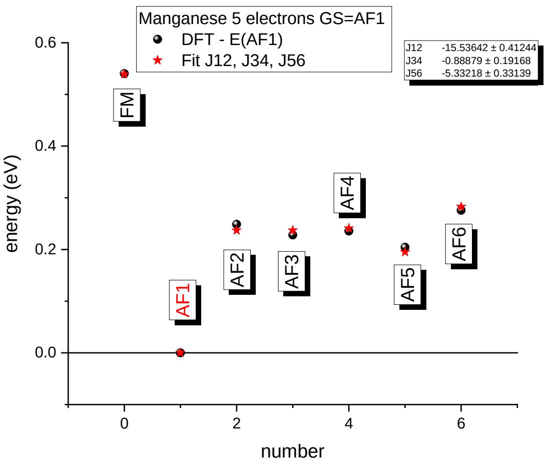

Figure S2. Results of the least-square fitting the relative energies of the seven ordered spin states (FM, AF1-AF6) of $MnPS_3$, determined by DFT+U calculations, in terms of the spin Hamiltonian defined by three exchange parameters J_{12}, J_{13} and J_{14}.

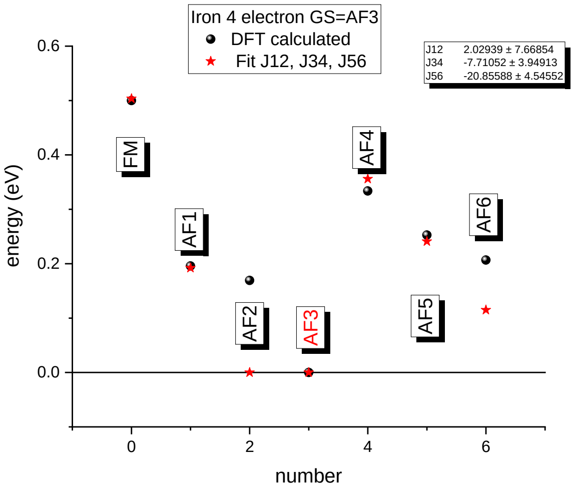

Figure S3. Results of the least-square fitting the relative energies of the seven ordered spin states (FM, AF1-AF6) of $FePS_3$, determined by DFT+U calculations, in terms of the spin Hamiltonian defined by three exchange parameters J_{12}, J_{13} and J_{14}.

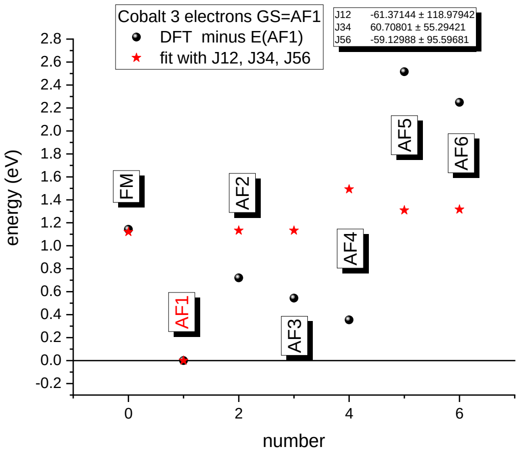

Figure S4. Results of the least-square fitting the relative energies of the seven ordered spin states (FM, AF1-AF6) of $CoPS_3$, determined by DFT+U calculations, in terms of the spin Hamiltonian defined by three exchange parameters J_{12}, J_{13} and J_{14}.

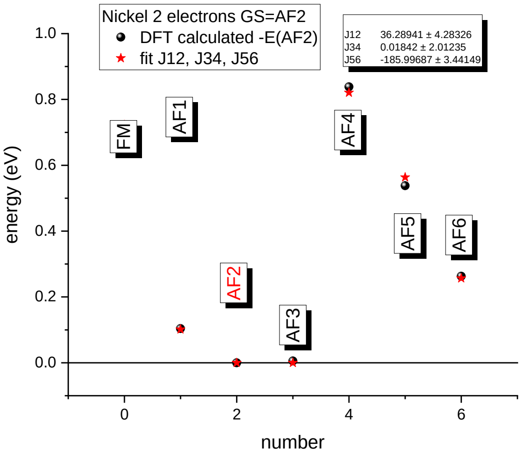

Figure S5. Results of the least-square fitting the relative energies of the seven ordered spin states (FM, AF1-AF6) of $NiPS_3$, determined by DFT+U calculations, in terms of the spin Hamiltonian defined by three exchange parameters J_{12}, J_{13} and J_{14}.

Table S1. Relative energies (in K per formula unit) of the ||x and ||z spin orientations obtained by MDD calculations for the M^{2+} ions of MPS_3 (M = Mn, Fe, Co, Ni) in the AFM1, AF2 and AFM3 states using the experimental crystal structures.

	||x	||z	||x	||z	||x	||z	||x	||z
AF1	0.48	0.17	0.33	0.11	0.20	0.07	0.09	0.03
AF2	0.00	0.35	0.00	0.24	0.00	0.14	0.00	0.06
AF3	0.55	0.38	0.38	0.26	0.22	0.15	0.10	0.07

Table S2. Spin exchanges (in K) obtained for the experimental structures of MPS_3 (M = Mn, Fe, Co, Ni) from DFT+U calculations with U_{eff} = 4 eV.

	Mn	Fe	Co	Ni
J_1	-16.05	-8.03	-121.59	40.28
J_2	-13.89	18.52	529.75)	22.67
J_3	-0.93	-8.98	294.51	-5.50
J_4	-0.81	-3.52	-2.74	1.30
J_5	-5.57	-13.55	-52.80	-181.66
J_6	-5.55	-30.66)	-549.38	-186.60
